# Socio-demographic factors related to periodontal status and tooth loss of pregnant women in Mbale district, Uganda

**DOI:** 10.1186/1472-6831-9-18

**Published:** 2009-07-18

**Authors:** Margaret Wandera, Ingunn MS Engebretsen, Isaac Okullo, James K Tumwine, Anne N Åstrøm

**Affiliations:** 1 Institute of Clinical Odontology, Faculty of Medicine and Dentistry, University of Bergen, Norway; 2 Department of Dentistry, Makerere University, Uganda; 3 Center for International Health, University of Bergen, Norway; 4 Department of Paediatrics & Child Health, School of Medicine, Makerere University, Kampala, Uganda

## Abstract

**Background:**

Information on the socio-behavioral distribution of periodontal status and tooth loss in pregnancy emanating from sub Saharan Africa is sparse. This study examined periodontal status and tooth loss in pregnant Ugandan women and assessed the relationship with socio-demographics factors, parity, dental care and oral hygiene.

**Methods:**

Mothers were participants of a multicentre cluster-randomized behavioral intervention study (PROMISE-EBF Safety and Efficacy of Exclusive Breast feeding Promotion in the Era of HIV in Sub-Saharan Africa). In Uganda, these were pregnant women resident in Mbale district, recruited into the PROMISE EBF study between January 2006 and June 2008. A total of 886 women were eligible to participate of whom information became available for 877 (participation rate 98.9%, mean age 25.6) women who participated in the recruitment interview and 713 (mean age 25.5) women who got a clinical oral examination. Periodontal status was assessed using the Community Periodontal Index of Treatment Needs (CPITN).

**Results:**

The prevalence of tooth loss was 35.7%, 0.6% presented with pockets shallow pockets (4–5 mm), whereas 3.3% and 63.4% displayed bleeding and calculus, respectively. A total of 32.7% were without any sign of periodontal disease. Binary logistic regression analyses revealed that older women, women from larger households and those presenting with microbial plaque were respectively, 3.4, 1.4 and 2.5 times more likely to have CPI score >0. Rural (OR = 0.9), nulliparous (OR = 0.4) and women who never visited a dentist (OR = 0.04) were less likely, whereas women from larger households (OR = 1.5) were more likely to have lost at least one tooth.

**Conclusion:**

The results revealed moderate prevalence of bleeding and tooth loss, high prevalence of calculus, low frequency of pockets 4–5 mm. Disparity in pregnant women's oral health related to parity suggests that education of maternity care providers concerning oral health in pregnancy is warranted.

**Trial registration:**

ClinicalTrials.gov Identifier NCT00397150

## Background

Studies using the Community Periodontal Index of Treatment Need (CPITN) have indicated that the prevalence of severe periodontal disease is low in sub-Saharan Africa [1-3]. However, with very few exceptions the oral hygiene condition has been described as poor with accumulation of plaque and calculus being more widespread with increasing age [2]. Previous reports considering the profile of periodontal status globally have concluded that the distribution of advanced periodontal destruction in adults is quite similar across populations in Africa (Kenya), Asia (Japan and China) America (Mexico) and Norway [4]. Recently, it was recognized that Black people are twice as likely as White people to have chronic periodontal problems, with males being most severely affected [5]. Exposure to risk factors, such as age, low socio-economic status, poor education, HIV infection, low dental care utilization, poor oral hygiene level, smoking, parity (i.e. number of children borne) and psycho social stress tend to concentrate in certain populations. These factors are more, or as important, as race and ethnicity [5-8]. Tooth loss is a final common pathway for oral diseases and is an important oral health indicator. It provides information as to the prevalence of oral diseases and may be an indication of the availability of dental care services. In sub-Saharan Africa, the prevalence of tooth loss (i.e. having lost at least one tooth due to any reason) is reported to range from 48% in Kenya to 96% in rural Tanzania [9]. Across East African countries women are more likely than men to experience tooth loss, although a recent survey of Tanzanian older adults revealed that females were more likely than males to have tooth loss due to caries whereas men were most likely to have tooth loss due to reasons other than caries [1,10].

Pregnancy affects a woman's hormonal exposure throughout life and hormonal exposure is related to elevated gingival inflammation, increased periodontal pocket depths and tooth loss [11-13]. The prevalence of gingivitis in pregnant women has reportedly ranged from 30% to 100% [13-15]. It is evident that periodontal disease in women of childbearing age remains prevalent, particularly among low-income women and members of racial and ethnic minority groups [16]. Since gingivitis is a known prelude to irreversible periodontal breakdown, repeated episodes of gingivitis during pregnancy might exacerbate chronic periodontal disease [17]. Thus, investigators continue to report higher prevalence of gingivitis in pregnant women compared with their non-pregnant counterparts [17]. However, the evidence on this topic is controversial and some studies have failed to demonstrate a correlation between pregnancy hormones, gingival inflammation and clinical attachment loss [15,18]. Moreover, it has been hypothesized that parity (i.e. number of births/live births) is associated with increased level of tooth loss. Few studies have, however, investigated this relationship focusing on pregnant women in low in come countries [11]. Information on the prevalence and social distribution of periodontal status and tooth loss in pregnancy emanating from sub Saharan African countries is sparse. A study of mothers and pregnant women in Dar es Salaam, Tanzania, reported an average of two teeth lost and a mean periodontal attachment loss of 3.1 mm [19]. This study showed a significant association between parity and periodontal attachment loss, but no relationship between parity and tooth loss [19]. Nuamah and Annan [20] examined Ghanaian pregnant women using the Community Periodontal Index of Treatment Need (CPITN), [21,22] and found a mean number of sextants with bleeding gingiva amounting to 0.69, 3.2 and 1.9 for non-pregnant women, pregnant second trimester and pregnant third trimester, respectively. To date, no studies have investigated the oral health status and its correlates among pregnant women in Uganda.

Adler et al. [23] have described a model suggesting that socio-economic position affects general health through health care, psycho-social factors and health related behaviors. Recently, Russell et al [24] used this model to explore the pathways between parity and tooth loss in women from the general US population. The present study aimed to examine the relationship of periodontal status and tooth loss in pregnant Ugandan women with parity, socio-economic factors, gestational age, dental care utilization and oral hygiene behavior. Following the propositions of Adler et al.'s model [23], it was assumed that socio-demographic position and parity (shown to be closely related to socio-economic status) affected tooth loss and periodontal problems in pregnant women independent of or through (i.e. mediated by) dental care utilization, psycho social factors and oral hygiene behavior.

## Methods

Participating women of the present study were members of a multicentre cluster-randomized behavioral intervention trial: Safety and Efficacy of Exclusive Breast feeding Promotion in the Era of HIV in Sub Saharan Africa – PROMISE EBF (Id NCT00397150 at ) conducted in Uganda, Burkina Faso, Zambia and South Africa. The aim of PROMISE EBF was to develop and test an intervention to promote exclusive breastfeeding, to assess its impact on infants in African contexts with a high prevalence of HIV and to strengthen the evidence base regarding optimal duration of exclusive breast feeding (EBF). In Uganda, Mbale district was purposively selected as the intervention site. The units for randomization were clusters made up of 1–2 villages with an average of 1000 inhabitants (35 infants per year given a birth rate of 3.5%). All pregnant women resident in twenty four clusters (18 rural and 6 urban), were eligible for the study. Clusters were selected according to accessibility in terms of being on a main road out from Mbale Municipality, having reasonable road standard during the rainy season, access to church, school, trading centre and water from the village cell. The women were recruited into the PROMISE EBF study between January 2006 and June 2008. There were a total of 6 interviews and one oral examination scheduled for each participant: a recruitment interview, oral health interview and a clinical oral examination during pregnancy, followed by interviews at 3-, 6-, 12-, and 24 weeks post partum. Women who did not intend to breastfeed and infants borne with serious diseases or deformities that prevent breastfeeding were excluded from participation. A total of 886 pregnant women were eligible to participate of whom information became available for 877 (participation rate 98.9%) (mean age 25.6, sd 6.4) women who participated in the recruitment interview and 713 (mean age 25.5 sd 6.6) women who underwent a clinical oral examination in their own homes. Reasons for not participating in the clinical examination were difficulties to locate women, withdrawal of consent and death. The number of participants satisfied a sample size of 800 pregnant women calculated for the oral sub-study, assuming a prevalence of tooth loss (i.e. at least one tooth lost) of 50%, a precision of 0.05 and a design effect of 2. The procedures of recruitment and participation are detailed in the PROMISE EBF study profile [25]. Ethical Clearance was obtained from the Ethical board, Faculty of Medicine, Makerere University. Written consent was obtained from all participants in the study and verbal consent prior to each examination and interview.

### Interviews

Structured interviews were designed with Epihandy software to be used on handheld computers. Interviews were conducted in face to face settings with participants at household level. The interview schedules were developed in English, translated into the local language of Lumasaaba and back translated into English. Health professionals reviewed the interview schedule for semantic, experiential and conceptual equivalence and sensitivity to culture and selection of appropriate words were considered. The interview schedules were piloted with 21 women-infant pairs attending the pediatric ward, Mbale Hospital, before administration. Adler et al.'s model [23], conceptualizing the relationship of health conditions with socio-economic status, health care, psycho social factors and health related behaviors was used to guide the identification of exploratory variables and the statistical analyses. *Socio-demographics *were assessed in terms of place of residence, age, wealth index, number of persons in the household, ownership of land, parity and use of bed nets (for malaria protection). Family wealth was assessed as an indicator of socio-economic status in accordance with a standard approach in equity analyses [26]. Household durable assets indicative of family wealth e.g. bicycle, television, car, motor cycle assessed as (1) available/in working condition, (2) not available/not in working condition were analyzed using principle component analysis. The first component resulting from the analysis was used to divide households into four approximate quartiles of wealth status ranging from 1^st ^quartile (least poor) to 4^th ^quartile (most poor). *Dental care *was assessed by one question considering time since last dental visit. *Psycho social factors *were assessed in terms of length of pregnancy, marital status and ever breast problem as a proxy of health problems in women [27]. Microbial plaque level was measured using the Oral Hygiene Index-Simplified (OHI-S) index by Greene & Vermillion [28] and used as a proxy of *oral hygiene behavior*. Debris was graded on a numeric scale from 0 to 3, divided by number of sites recorded and categorized in terms of low debris (0) (score 0.0–0.67) and fair debris (1) (score 0.68–1.67). Socio-economic and behavioral variables used as explanatory variables in the analyses, their coding and the number of subjects (%) according to categories in urban and rural areas is depicted in Table 1.

**Table 1:** Frequency distribution of participants in urban and rural areas according to category of independent variables (n = 877)

		Urban		Rural		p-value
		%	(n = 234)*	%	(n = 633)*	
Age:	≤ 20 yr	28.4	(63)	25.6	(158)	
	21–30 yr	59.0	(131)	51.9	(320)	
	31–45 yr	12.6	(28)	22.5	(139)	.006
Education:	Low	14.5	(31)	21.6	(122)	
	Medium	55.6	(119)	65.2	(369)	
	High	29.9	(64)	13.3	(75)	.000
Household assets:	1^st ^quartile-most poor	12.0	(26)	21.4	(130)	
	2^nd ^quartile	22.7	(49)	40.7	(247)	
	3^rd ^quartile	18.1	(39)	20.4	(124)	
	4^th ^quartile – least poor	47.2	(102)	17.5	(106)	.000
Number in household:	1–4	64.7	(145)	52.2	(325)	
	5–20	35.3	(79)	47.8	(298)	.001
Household owns land:	No	77.9	(158)	22.7	(141)	
	Yes	29.1	(65)	77.3	(479)	.000
Marital status:	Not married	48.7	(109)	34.7	(217)	
	Married	51.3	(115)	65.3	(409)	.000
Last dental visit:	less than 6 months ago	8.3	(17)	4.7	(28)	
	more than 6 months	28.9	(59)	22.5	(134)	
	never	62.7	(128)	72.8	(433)	.016
Months of pregnancy:	seven or more	87.5	(196)	83.4	(497)	
	less than seven	12.5	(28)	16.6	(99)	.160
Parity:	One child or more	74.6	(167)	78.3	(490)	
	None	25.4	(57)	21.7	(136)	.147
Ever breast problems:	No	81.4	(179)	80.9	(501)	
	Yes	18.6	(41)	19.1	(118)	.921
Use of bed nets:	No	30.0	(67)	57.4	(353)	
	Yes	70.0	(156)	42.6	(262)	.000
Debris:	low	85.6	(155)	78.9	(416)	
	fair	14.4	(26)	21.1	(111)	.050

*The number of participants in different categories do not add up to 234 in urban and 633 in rural due to missing responses

### Clinical oral examination and outcome variables

A trained and calibrated dentist (MW) carried out all clinical oral examinations under field conditions based on the World Health Organization (WHO) criteria [22], recording the data on a prepared record sheet. All fully erupted permanent teeth were scored excluding third molars. Oral examinations were performed at women's household with subjects seated, and the examiner used a headlamp as source of illumination, mouth mirror and a CPITN periodontal probe in line with the WHO instructions. *The periodontal status was *assessed using a specially designed lightweight CPITN probe with a 0.5 mm ball tip. Periodontal pockets were measured from the edge of the free gingiva to the bottom of the pocket. Using the epidemiological part of the CPITN, the Community Periodontal Index (CPI) [21,22] with 10 index teeth (17,16,11,26,27,47,46,31,36,37) and 6 sextants (17–14, 13–23, 24–27, 38–34, 33–43, 44–47) per individual, four indicators of periodontal status were applied. Only index teeth were examined according to the following criteria; healthy periodontal status (code 0), bleeding on probing observed (code 1), calculus detected during probing (code 2), pocket 4–5 mm (code 3) and pocket ≥ 6 mm (code 4). Each index tooth was scored on 2 sites (buccal and lingual) and each sextant was scored according to its highest CPI score. If no index tooth was present in a sextant, all the remaining teeth in that sextant were examined and the highest score is recorded as the score for that sextant. In accordance with the hierarchical assumption, teeth with score 3 were assumed positive with respect to bleeding and calculus whereas teeth with score 2 were assumed positive with respect to bleeding [3]. Prevalence of bleeding-, calculus and pocket sextants was assessed as the percentage of subjects affected. Prevalence of healthy sextants was assessed as the number of subjects having 6 healthy sextants. Severity of periodontal condition was assessed by the mean number of sextants having CPI code 0, 1 or higher, 2 or higher, 3 or higher and 4. Total CPI was presented as the percentage distribution of dentate subjects according to the highest score in the mouth [21]. *Tooth loss due to any reason *was recorded as absent (0) and present (1) for all teeth except the third molars.

### Reproducibility

Duplicate clinical examinations were carried out with 50 mothers considered to be representative of the study participants and after a period of one month. Analysis performed on the duplicate examination recordings gave Kappa values of 0.91 for missing teeth. With respect to indicators of periodontal condition, kappa values ranged from 0.48 (CPI index tooth 11) to 0.85 (CPI index tooth 31). These figures indicate moderate to good intra examiner reliability [29].

### Statistical analysis

Data was entered into Epihandy program on the handheld computers and analyzed using SPSS version 15.0 (Chicago, IL, USA). Cross tabulation, chi square statistics and Univariate ANOVA were used to assess bivariate relationships. Multiple logistic regression analyses were conducted with CPI score >0 and missing teeth as dependent variables using the logit model and 95% Confidence intervals (CI) given for the odds ratios.

## Results

### Description of the study population

As shown in Table 1, a total of 26.7% of the participants were resident in urban areas while 73.3% were from the rural areas of Mbale district. The majority were in or beyond their 7 month of gestation. Only 2.7% of the women confirmed to use any kind of tobacco product. The frequency distribution of socio-demographic characteristics varied systematically with place of residence. Urban women were younger, had higher level of education, were less poor according to the wealth index, more often unmarried, more often dental visitors, used bed nets more frequently and presented less often with bad oral hygiene as compared to their rural counterparts.

### Non response analyses

One hundred and sixty four out of the 877 interviewed women did not undergo clinical oral examination. In order to examine the possibility that selection bias occurred from this sample attrition, a comparison was made of the socio-demographic characteristics of participants (n = 713) and non-participants (n = 164) of the oral clinical examination. The results revealed less substantial differences between the two groups with the frequency distributions of age, education, household assets and parity for the two groups being similar. However, 78% versus 68% (p < 0.05) of non-respondents and respondents respectively, had never visited a dentist (see additional file 1).

### Prevalence of periodontal status and tooth loss

**Figure 1:**
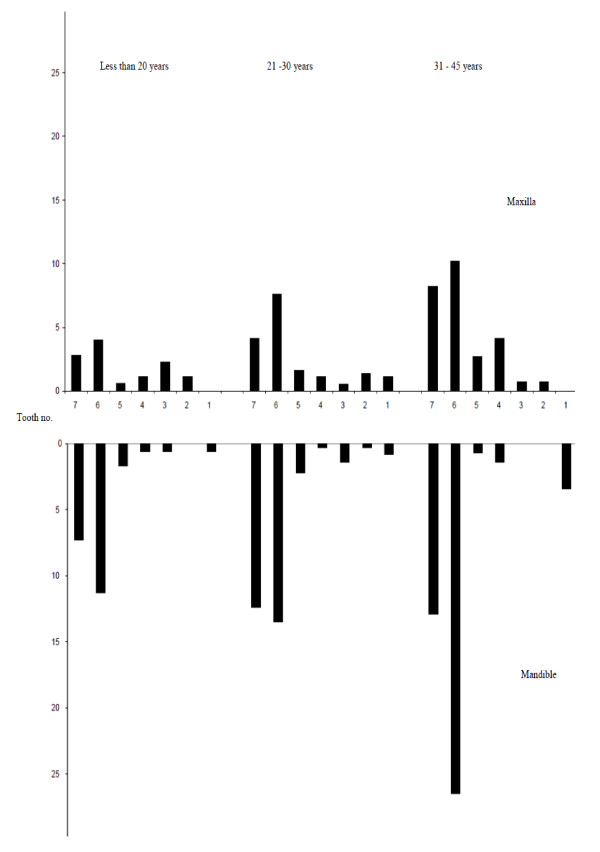
**Percentage of tooth loss by tooth type and age group**. Tooth number illustration: 1: Central Incisor, 2: Lateral Incisor, 3: Canine, 4: First premolar, 5: Second premolar, 6: First Molar, 7: Second molar.

The prevalence of tooth loss (≥ 1 tooth lost due to any reason) was 35.7% (42.5% in urban and 33.8% in rural areas, p < 0.05). Direct age standardization did not alter the crude urban rural difference in the prevalence of tooth loss. Participants from the urban areas had on average lost 0.79 (sd = 1.2) teeth, whereas their rural counterparts had lost 0.75 (sd = 1.3) teeth (n.s). The mean debris score according to the OHI-S index was 0.38 (sd = 0.35). The distribution of tooth loss due to any reason according to tooth type and age groups is depicted in Figure 1. The proportion of subjects having CPI codes of 0, 1, 2 and 3 as their highest individual score were respectively, 37.0%, 4.4%, 56.9% and 1.7% in the urban and 31.7%, 2.8%, 65.3% and 0.2% in the rural area. Direct age standardization did not alter the urban rural differences in crude total CPI scores. Table 2 depicts the prevalence of subjects with CPI score 0, 1, 2 and 3 and the mean number of sextants with CPI score 0, CPI score 1 or higher and CPI score 2 or higher according to age as recommended by WHO [22]. The prevalence of subjects having CPI score 0 and mean number of healthy sextants decreased with increasing age. The prevalence of subjects with score 1 decreased with increasing age, whereas prevalence of subjects with CPI score 2 and 3 and the prevalence of tooth loss increased with increasing age. Mean number of sextants with healthy periodontal tissue (CPI = 0), decreased with increasing age whereas mean number of sextants with bleeding or higher and calculus or higher increased with increasing age.

**Table 2:** Percentage (n) and mean number (95% CI) of periodontal parameters corresponding to CPI scores; 0, 1, 2 and 3

Age	Number of sextants CPI = 0	CPI = 0	Number of sextants CPI = 1 or higher	CPI = 1	Number of sextants CPI = 2 or higher	CPI = 2	CPI = 3
	Mean (95% CI)	% (n)	Mean (95% CI)	% (n)	Mean (95% CI)	% (n)	% (n)
15–20	5.0 (4.8–5.1)	48.0 (85)	1.0 (0.8–1.1)	6.8 (12)	0.8 (0.6–0.9)	44.6 (79)	0.6 (1)
21–30	4.6 (4.4–4.7)	31.6 (117)	1.3 (1.2–1.5)	3.0 (11)	1.2 (1.2–1.4)	65.1 (241)	0.3 (1)
31–45	3.9 (3.7–4.2)	17.0 (25)	1.9 (1.7–2.2)	0.0 (0)	1.8 (1.5–2.1)	81.6 (120)	1.4 (2)
Total	4.5 (4.4–4.6)**	32.7 (227)	1.4 (1.2–1.5)**	3.3 (23)	1.2 (1.2–1.3)**	63.4 (440)	0.6 (4)

** p < 0.001, *p < 0.05

### Correlates of periodontal status and tooth loss

Table 3 shows indicators of periodontal disease in terms of prevalence of subjects having CPI >0 and prevalence of tooth loss as related to socio-demographic-, dental care-, psycho-social-and oral hygiene characteristics. The prevalence of subjects having CPI score 3 (4–5 mm pockets) was low in this study population (0.6%) and was excluded from further analysis because of the estimates being subject to large random variation. As shown, socio-demographics in terms of age, number of members in household, use of bed net and parity were statistically significantly associated with having CPI score >0 in addition to marital status and oral hygiene (Table 3). All variables that were statistically significantly associated with CPI >0 in the bivariate analyses were entered into a hierarchical binary multiple logistic regression models. According to Table 3, the adjusted ORs for having CPI score >0 were 1.7 and 3.4 in middle aged and older women, respectively as compared to younger women, 1.4 in women from larger compared to smaller households, 0.6 in women using bed nets as compared to their counterparts who did not and 2.5 in women with bad compared to women with good oral hygiene. Prevalence of toothloss was statistically significantly associated with place of residence, wealth index, age, size of household, parity, use of bed nets, dental visits and breast problems. In the final stage of the multiple logistic regression analyses, size of household, parity, dental visits and breast problems remained statistically significantly associated with tooth loss. The corresponding ORs were 1.5, 0.4, 0.04 and 0.3, respectively.

**Table 3:** Indicators of pregnant women's oral condition using percentages (n) of subjects having CPI score >0 and ≥ 1 missed tooth.

		*CPI >0 unadj*		*CPI >0 adjusted*^±^**§**		*≥ 1 missed tooth unadjusted*		*≥ 1 missed tooth Adjusted***§**	
**SES**		%	(n)	OR	95% CI	%	(n)	OR	95% CI
Place of residence:	urban	63.0	114			42.5	77	1	
	rural	68.3	360			33.8	349*	0.9	0.5–1.6
Wealth index:	1^st ^quartile	68.5	85			29.0	36	1	
	2^nd ^quartile	66.9	164			31.0	76	0.9	0.5–1.7
	3^rd ^quartile	73.4	102			42.4	59	1.2	0.6–2.6
	4^th ^quartile	63.4	111			45.1	79*	1.1	0.5–2.2
Age:	≤ 20 yr	52.0	92	1		26.6	47	1	
	21–30 yr	68.4	253	1.7	1.2–2.7	37.0	137	0.8	0.4–1.5
	31–45 yr	83.0**	122	3.4	1.8–6.1	43.5	64*	1.0	0.5–2.1
Household	1–4 persons	61.1	240	1		30.3	119	1	
	5–20 persons	78.8**	231	1.4	1.0–2.0	43.0	133**	1.5	1.0–2.5
Owning land:	No	64.8	162			34.8	87		
	yes	68.3	306			36.8	165		
Parity:	At least one	70.9	389	1		40.4	222	1	
	Never	53.5**	83	0.7	0.4–1.1	19.4	30**	0.4	0.2–0.8
Use bed net:	No	72.1	248	1		32.0	110	1	
	Yes	62.1*	218	0.6	0.4–0.9	40.2	141*	0.9	0.5–1.4
**Dental care**									
Last dental visit:	<6 months	71.4	25			82.9	29	1	
	>6 months	67.0	118			77.3	136	0.7	0.2–2.0
	Never	67.0	308			15.4	71**	0.04	0.02–0.1
**Psycho-social**									
Pregnancy:	≥ 7 month	66.3	375			37.3	211		
	< 7 month	70.5	74			29.5	31		
Marital status:	Not married	62.3	160	1		31.9	82		
	married	69.8*	312	0.9	0.6–1.4	38.0	170		
Breast Problem	No	66.5	374			37.9	213	1	
	Yes	68.1	92			27.4	37*	0.3	0.2–0.6
**Oral hygiene**									
Debris:	low	63.3	364	1		37.0	213		
	fair	82.6**	114	2.5	1.5–4.1	31.9	44		

** p < 0.001, *p < 0.05

^±^Adjusted for number of teeth lost

§Only variables that were statistically significantly associated with dependent variables were entered into the regression models

## Discussion

In accordance with the propositions of Adler et al. [23] and consistent with previous findings from industrialized countries [24], parity was positively associated with the prevalence of periodontal disease and toothloss among pregnant women in Mbale. Only the latter relationship maintained statistical significance after having adjusted for relevant covariates. Thus, the effect of parity on the prevalence of periodontal disease might have been confounded or mediated by other socio-demographic variables, psycho-social- and oral hygiene related factors. Notably, this study does not address any mechanism by which pregnancy related factors may adversely influence women's oral health condition. Confounding related to biological and behavioral factors in common might be alternatives to a biological explanation of those relationships.

Studies of periodontal condition vary considerably with respect to ethnicity and age of the population considered. A range of various definitions of periodontal disease in terms of gingival bleeding, probing pocket depths, loss of attachment and radiographic bone loss have been utilized [2]. In addition, there are considerable variations in the number of sites per tooth and number of teeth examined [5,30]. This inconsistency in methodology and use of disease parameters influences results and limits valid comparisons between studies. Many studies have shown that prevalence and severity estimates as well as the distributional characteristics of periodontal condition vary depending on the method used for recording [2,3,15,30-32]. The present study population was quite homogeneous with respect to ethnicity and the restricted age range (15–45 years) limited to some extent the confounding effect of age related factors. Since the prevalence of subjects who used any tobacco products was negligible, the confounding and possible modifying effect of smoking, considered to be a risk factor for periodontal disease was limited, as well [33].

Despite its methodological limitations, the epidemiological part of the CPITN, the CPI, [21,22] was deemed an appropriate screening system for the present study, considering that clinical examinations were carried out under field conditions in household settings. CPI includes all periodontal disease indicators from bleeding on probing (code 1) to advanced periodontal disease (code 4) and has been used extensively in various populations, in Europe and Africa, such as for instance Kenya, Tanzania and Ethiopia [2]. This index does not make a distinction between gingival inflammation and periodontal destruction due to its hierarchical scoring principle [31,32]. Grytten et al [34] showed, however, that close to 30% of teeth with calculus do not represent bleeding and that one fourth of teeth with deep pockets and bleeding do not present with calculus. CPI scores 1 and 2 reflect gingival inflammation and poor oral hygiene, conditions that are common but does not necessarily progress to periodontal destruction [30]. Other shortcomings are that measures of clinical attachment loss and tooth mobility are not considered. Finally, the use of index teeth, instead of a full mouth recording has been shown to increase underestimation of the prevalence of periodontal pockets [2].

Keeping in mind the limitations associated with CPI and that this method does not constitute a complete measure of periodontal conditions, this study indicated absence of severe periodontal disease in terms of CPI code 4 and a very low prevalence of pockets of 4–5 mm corresponding to CPI score 3. As shown in Table 2, only 0.6% of the women investigated presented with CPI score 3. In contrast, 3.3% and 63% had bleeding and calculus, respectively. The overall picture of the periodontal condition observed, characterized by low to moderate prevalence of bleeding of low severity, by high prevalence of light calculus deposits and by infrequent occurrence of shallow pockets, 4–5 mm is consistent with CPITN based findings in the African populations generally [2]. Using number of subjects with bleeding, calculus and pockets as periodontal outcome variables instead of for instance alveoloar bone- and attachment loss focuses the extent of the infection at the time of the survey rather than on consequences of past disease processes. Direct comparison of the present prevalence estimates with that found in other countries is of limited value due to the variations in scoring between investigators. Davenport et al. [35] reported on a prevalence of pockets (i.e. CPITN 4) of 44% in a group of UK women of various ethnicity assessed immediately after delivery. Miyazaki et al [36] in a study of pregnant Japanese women reported a prevalence of 31% pockets of 4–5 mm. In the present study, 67% of the pregnant women presented with any sign of periodontal disease (CPI >0). The corresponding finding in Japan was 97% [36]. Moreover, the mean number of bleeding sextants or higher, ranging from 1.0 in the youngest to 1.9 in the oldest age group is considerably lower and in line with corresponding figures of 3.2 and 1.9 observed in pregnant Ghanaian women during their second and third trimester, respectively [20]. Pregnant Ugandan women had lost on average 0.7 teeth which is considerably lower than the 2 teeth lost per individual reported among pregnant women of similar age in Tanzania [19].

Older women, women of lower socioeconomic status in terms of larger households (lower family size often reflect higher level of female education) and those having poor oral hygiene were more likely than their counterparts to present with any sign of periodontal disease. In contrast, women who confirmed use of bed net recognized as a proxy of social status and positive health attitudes [37], were less likely to present with any sign of periodontal disease. A pattern of positive correlation between periodontal disease and age has been found in numerous studies globally, such as for instance among Ugandan students where similar age distributions were observed regarding aggressive- as well as chronic periodontal disease [38]. The present findings accord with a strong social gradient in periodontal disease confirmed earlier in pregnant women as well as in adolescents and adults from the general population globally [39,40]. Thus, contemporary evidence suggest that the lower the material standard of living, the worse the periodontal status irrespective of the measure (being it clinically assessed or self reported oral health) used to assess it. Based on data from the 1999–2004 National Health and Examination Nutrition Surveys, Borell and Crawford [8] concluded that inequalities in periodontal health associated with race/ethnicity, education and income continues to be pervasive in the US population over years, whereas the disease difference between low and high educated individuals appears to have increased during the last 10 years.

Contrary to what was hypothesized [41], gestational age did not influence periodontal status among the pregnant women investigated in this study. In a recent longitudinal study of pregnant women in Finland, bleeding on probing increased between the first and second trimester, without relation to the plaque level recorded [15]. Studies from Sri Lanka have presented similar results with higher proportions of women having at least one sextant with bleeding during the third than during the second trimester [18]. In general, the impacts assessed in this study of parity, socio-demographics and gestational age on the periodontal condition of pregnant women was limited compared to the importance of oral hygiene and age. However, all attributes investigated in terms of age, socio-demographics, oral hygiene, parity and month of pregnancy might be considered risk indicators for periodontal disease in pregnant women. Previous studies have shown that the mean plaque index in smokers is double that of non smokers and that oral hygiene measures mediate the effect of smoking on the periodontal condition [42]. Oral hygiene presented as a strong correlate of periodontal indicators in pregnant non-smoking Ugandan women and the mean plaque level recorded was low. This might indicate that there might be minor effects if any due to social desirability in terms of women taking the opportunity to brush in advance of the clinical examination at household level.

Ugandan women of higher parity seemed to be more likely to present with tooth loss than were their counterparts (Table 3). This could be attributed to incomplete control of utilization of dental care and socio-demographic variables in the analyses, alternatively the relationship could be interpreted as accumulated tissue destruction across time rather than an intrinsic parity related abnormality. In fact, previous studies have shown that both pregnancy and maternity alter dental visiting-and treatment patterns in women [43]. Although a minority of the pregnant women investigated in this study had their last dental visit less than 6 months ago (Table 1), frequent dental attendees presented with a higher prevalence of tooth loss than their non visiting counterparts. This study is the first to confirm the hypothesis that parity is positively related to number of missing teeth in young pregnant low-income country women. Previous studies have investigated women after delivery and some have used data on elderly Scandinavian women [44]. Scheutz et al. [19] reported on no association between parity and tooth loss, whereas attachment loss was found to be more pronounced in multiparous than in nulliparous women.

The present study was based on recruited women in PROMISE-EBF [25] and limited by the sampling methodology used in the main study. Thus the representativeness of the study participants with respect to the population of pregnant women in Mbale district or in Uganda as a whole cannot be asserted. However, the main aim of the present study was to identify correlates of periodontal disease and tooth loss in pregnancy and to examine the pathways of association of socio-demographics, parity and health related variables with the dependent variables of interest. As such the external validity was deemed to be of less importance.

## Conclusion

The oral condition of pregnant women was characterized by low prevalence of bleeding, high prevalence of calculus deposits, low prevalence of 4–5 mm pockets and by a moderate prevalence of tooth loss. Age, social status, oral hygiene and parity might be potential risk factors for chronic periodontal disease in this study population. Additional research is needed to determine the nature of those relationships. Disparity in pregnant women's oral health related to parity suggests that education of maternity care providers concerning oral health in pregnancy is warranted.

## Competing interests

The authors declare that they have no competing interests.

## Authors' contributions

All authors participated in the design and planning of the study: MW: conducted field work, statistical analyses and manuscript writing. ANÅ: main supervisor, conducted statistical analyses, and manuscript writing. IMSE: contributed to manuscript writing. IO and JKT: contributed in data collection supervision and have been involved in revising manuscript. All authors read and approved the final manuscript.

## Pre-publication history

The pre-publication history for this paper can be accessed here:



## Supplementary Material

Additional file 1 **Supplemental Table**. Comparison of participants (n = 713) and not participating (n = 164) subjects in oral clinical examination. Click here for file
