# Supplementary material for: Socio-demographic factors related to periodontal status and tooth loss of pregnant women in Mbale district, Uganda
Source: BMC Oral Health. 2009 Jul 18;9:18. doi: 10.1186/1472-6831-9-18 (PMC2722607; doi:10.1186/1472-6831-9-18)
Supplement: Additional file 1 — Supplemental Table. Comparison of participants (n = 713) and not participating (n = 164) subjects in oral clinical examination. [file 1472-6831-9-18-S1.doc]

Table 2. Comparison of participants (n=713) and not participating (n=164) subjects in oral clinical examination

|  |  | Not participating  % (n=164) | | Participating  % (n=713) | | p-value |
| --- | --- | --- | --- | --- | --- | --- |
| Age: | ≤ 20 yr | 30.2 | ( 45) | 22.5 | (177) |  |
|  | 21-30 yr | 56.4 | ( 84) | 53.3 | (370) |  |
|  | 31-45 yr | 13.4 | ( 20) | 21.2 | (147) | .083 |
| Household assets: | 1^st^ quartile-most poor | 23.1 | ( 33) | 18.2 | (124) |  |
|  | 2^nd^ quartile | 36.4 | ( 52) | 35.9 | (245) |  |
|  | 3^rd^ quartile | 17.5 | ( 25) | 20.4 | (139) |  |
|  | 4^th^ quartile – least poor | 23.1 | ( 33) | 25.6 | (175) | .511 |
| Education: | Low | 19.1 | ( 25) | 19.6 | (128) |  |
|  | Medium | 64.1 | ( 84) | 62.3 | (407) |  |
|  | High | 16.8 | ( 22) | 18.1 | (118) | .918 |
| Months of pregnancy: | seven or more | 85.6 | (131) | 84.4 | (566) |  |
|  | less than seven | 14.4 | ( 22) | 15.6 | (105) | .804 |
| Parity: | one or more | 74.0 | (111) | 78.0 | (549) |  |
|  | none | 26.0 | ( 39) | 22.0 | (155) | .285 |
| Last dental visit: | less than 6 months ago | 7.4 | ( 10) | 5.2 | ( 35) |  |
|  | more than 6 months | 14.1 | ( 19) | 26.2 | (176) |  |
|  | never | 78.5 | (106) | 68.6 | (460) | .009 |
| Place of residence: | Urban | 32.1 | ( 50) | 25.6 | (181) |  |
|  | Rural | 67.9 | (106) | 74.4 | (527) | .110 |
